# Solitary fibrous tumor of the oral cavity: a systematic review of the literature and a new clinicopathologic case report

**DOI:** 10.3389/froh.2026.1849788

**Published:** 2026-06-30

**Authors:** Fabio Maglitto, Alessio Danilo Inchingolo, Stefan Cocis, Chiara Copelli, Grazia Marinelli, Francesca Calò, Claudia Ciocia, Antonio Rizzo, Francesco Inchingolo, Andrea Palermo, Marco Severino, Angelo Michele Inchingolo, Gianna Dipalma

**Affiliations:** 1 Maxillofacial Surgery Unit, Department of Neurosciences, Reproductive and Odontostomatological Sciences, University Federico II, Naples, Italy; 2 Interdisciplinary Department of Medicine, School of Medicine, University of Bari “Aldo Moro”, Bari, Italy; 3 Interdisciplinary Maxillofacial Surgery Unit, Department of Medicine, School of Medicine, University of Bari “Aldo Moro”, Bari, Italy; 4 Department of Experimental Medicine, University of Salento, Lecce, Italy; 5 Department of Medicine and Surgery, University of Perugia, Perugia, Italy; 6 Department of Biomedical, Surgical and Dental Sciences, University of Milan, Milan, Italy

**Keywords:** immunohistochemistry, oral cavity, palate, solitary fibrous tumor, spindle-cell tumors

## Abstract

**Background:**

Solitary fibrous tumor (SFT) is a rare mesenchymal neoplasm of fibroblastic differentiation, originally described in the pleura and subsequently documented at multiple extrapleural sites. Oral involvement is uncommon and may be diagnostically challenging because of its variable clinical presentation and overlapping spindle-cell morphology.

**Materials and methods:**

We report a palatal SFT with clinical, imaging, histopathological and immunohistochemical correlation. In parallel, a systematic review was conducted in PubMed, Scopus and Web of Science for studies published between January 2015 and December 2025. Case reports, case series and retrospective clinicopathologic studies describing primary oral SFT were included. Study selection followed PRISMA 2020 recommendations and methodological quality was appraised qualitatively using Joanna Briggs Institute (JBI) critical appraisal tools appropriate to each study design.

**Results:**

Twelve eligible studies were included, comprising 10 case reports and 2 retrospective series for a total of 30 previously published oral SFTs. Soft-tissue lesions predominated, and the buccal mucosa/cheek was the most common location, followed by the floor of the mouth, tongue, labial mucosa, retromolar pad and intraosseous mandible. Across the reviewed studies, oral SFTs typically presented as slow-growing, well-circumscribed nodules and showed a patternless spindle-cell proliferation in a collagenous to hyalinized stroma with branching staghorn-like vessels. Signal Transducer and Activator of Transcription 6 (STAT6) was the most informative confirmatory marker, while Cluster of Differentiation 34 (CD34), B-cell Lymphoma 2 (BCL2) and Cluster of Differentiation 99 (CD99) were supportive but less specific. Most lesions were managed by complete local excision and showed a favorable course. In the present case, diffuse nuclear STAT6 positivity together with CD34, CD99 and BCL2 expression supported the diagnosis of palatal SFT.

**Conclusion:**

Oral SFT is an uncommon but distinctive fibroblastic neoplasm that should be included in the differential diagnosis of spindle-cell lesions of the oral cavity. Diagnosis relies on integration of morphology with immunohistochemistry, particularly STAT6. Complete surgical excision with margin assessment remains the cornerstone of treatment, and a risk-adapted long-term follow-up strategy is advisable because rare recurrences may occur, particularly in intraosseous, margin-positive, or proliferatively active tumors.

**Systematic Review Registration:**

The systematic review was registered on Prospero with code ID 1358544

## 1 Introduction

Solitary fibrous tumor (SFT) is a rare mesenchymal neoplasm characterized by fibroblastic differentiation (1–4). Although originally described in the pleura in 1931, it is now recognized as a distinct soft-tissue tumor that can arise in a broad range of extrapleural locations, including the head and neck region (5, 6). Oral SFT is uncommon and usually presents as a slow-growing, well-circumscribed submucosal mass covered by intact mucosa (7–10). Because its clinical appearance is nonspecific, the lesion may be mistaken for salivary gland tumors, vascular lesions, or other benign mesenchymal proliferations (11–14). Histopathologically, SFT is characterized by variable combinations of patternless spindle-cell proliferation, collagenous to hyalinized stroma, and thin-walled branching vessels with a staghorn-like configuration (15–18). These findings are characteristic but not pathognomonic, making immunohistochemistry essential in the differential diagnosis (19–21). Among ancillary markers, CD34, BCL2 and CD99 are frequently positive but lack specificity (22, 23). By contrast, nuclear STAT6 expression, which reflects the NGFI-A-Binding Protein 2 (NAB2)-STAT6 fusion, is currently considered the most useful immunohistochemical marker for confirming SFT in routine practice (24–27). Although many oral SFTs behave indolently, recurrence has been reported, particularly in cases with incomplete excision or unusual intraosseous presentation (28–30). The aim of the present study was therefore twofold: to describe a new palatal SFT with clinicopathologic correlation and to synthesize the recent literature on oral SFT through a systematic review (31–33). Given the rarity of palatal involvement, additional well-documented cases may help refine the clinicopathological spectrum of oral SFT (34–37).

## 2 Materials and methods

This study presents a clinical case report of a palatal SFT combined with a systematic review of the literature. The clinical case is reported in accordance with the CARE (CAse REports) guidelines, while the literature review was structured and reported according to the Preferred Reporting Items for Systematic Reviews and Meta-Analyses (PRISMA 2020) statement. It was registered in the International Prospective Register of Systematic Reviews (PROSPERO), under registration number ID 1358544. Because the available evidence consisted mainly of case reports and case series, the synthesis was descriptive and no meta-analysis was attempted. The systematic review component included only previously published data, whereas the original unpublished data were limited to the newly reported clinicopathologic case, for which written informed consent and institutional ethical approval were obtained.

### 2.1 Eligibility criteria

The review question was: “What are the clinicopathologic features, immunohistochemical characteristics, management strategies and outcomes of SFT arising in the oral cavity?”

Eligible studies included case reports, case series, and retrospective clinicopathologic studies describing primary oral SFT involving the buccal mucosa, palate, tongue, floor of the mouth, retromolar region or intraosseous mandible. Only human studies published in English between January 2015 and December 2025 were considered.

### 2.2 Inclusion criteria

Three reviewers assessed potentially eligible studies according to the following inclusion criteria:

1. Human subject studies involving patients with primary oral SFT.
2. Full-text articles available in English.
3. Studies published between January 2015 to December 2025.
4. Case reports, case series, and clinicopathologic studies.
5. Primary SFT localized within the oral cavity (e.g., palate, buccal mucosa, tongue, floor of the mouth or mandible).
6. Cases with a diagnosis of SFT supported by appropriate histopathological and immunohistochemical and/or molecular findings.

### 2.3 Exclusion criteria

1. Articles written in languages other than English.
2. Off-topic studies, including SFTs located in extra-oral sites (e.g., pleura, orbit, or skin).
3. Studies focused on other spindle-cell tumors (e.g., schwannoma, leiomyoma, myofibroma) without a confirmed SFT diagnosis.
4. *In vitro* studies, animal models, narrative reviews, and letters to the editor without original case data.
5. Studies with insufficient clinicopathological or follow-up data.

### 2.4 Search strategy

A comprehensive literature search was performed in PubMed, Scopus, and Web of Science. The search strategy combined terms related to solitary fibrous tumor with oral-site keywords and was adapted to the syntax of each database. In PubMed, the following search string was used: (“solitary fibrous tumor” OR “solitary fibrous tumour” OR hemangiopericytoma OR haemangiopericytoma) AND (“oral cavity” OR mouth OR palate OR tongue OR “floor of mouth” OR “buccal mucosa” OR cheek OR mandible OR gingiva OR retromolar). Equivalent search strings were adapted for Scopus and Web of Science using title, abstract, and keyword fields. The search window covered articles published from January 2015 to December 2025. The search was supplemented by manual screening of the reference lists of eligible studies to identify additional relevant reports. Duplicate records were removed before title and abstract screening.

### 2.5 Study selection and data extraction

Two reviewers independently screened titles and abstracts and subsequently assessed full texts for eligibility. Disagreements were resolved by discussion and persistent disagreements were resolved by consultation with a third reviewer. Data extraction was performed using a standardized data collection form.

The following data were extracted from each included study: publication year; study design; number of patients; oral site involved; major clinical features; imaging findings when available; key histopathological findings; immunohistochemical markers; Ki-67 proliferation index and/or mitotic activity when reported; treatment modality; surgical margin status when available; follow-up duration; recurrence and other risk-related features.

### 2.6 Quality assessment

Two reviewers independently appraised the methodological quality of the included studies using the Joanna Briggs Institute (JBI) critical appraisal tools appropriate to each study design. The JBI Checklist for Case Reports was applied to single case reports, whereas the JBI Checklist for Case Series was used for retrospective series. The appraisal focused on completeness of clinical description, diagnostic work-up, treatment reporting, follow-up and outcome reporting. Disagreements were resolved by discussion and, when necessary, by consultation with a third reviewer.

### 2.7 Ethical considerations

The clinical component of the study was conducted in accordance with the Declaration of Helsinki. Written informed consent for publication of clinical, radiological and histopathological material was obtained from the patient. The case report formed part of an institutional observational study approved by the Institutional Review Board of the University of Bari “Aldo Moro” (Prot. No. 0029116, 23 March 2023; study code: EAPAS).

## 3 Results

### 3.1 Study selection

**Figure 1:**
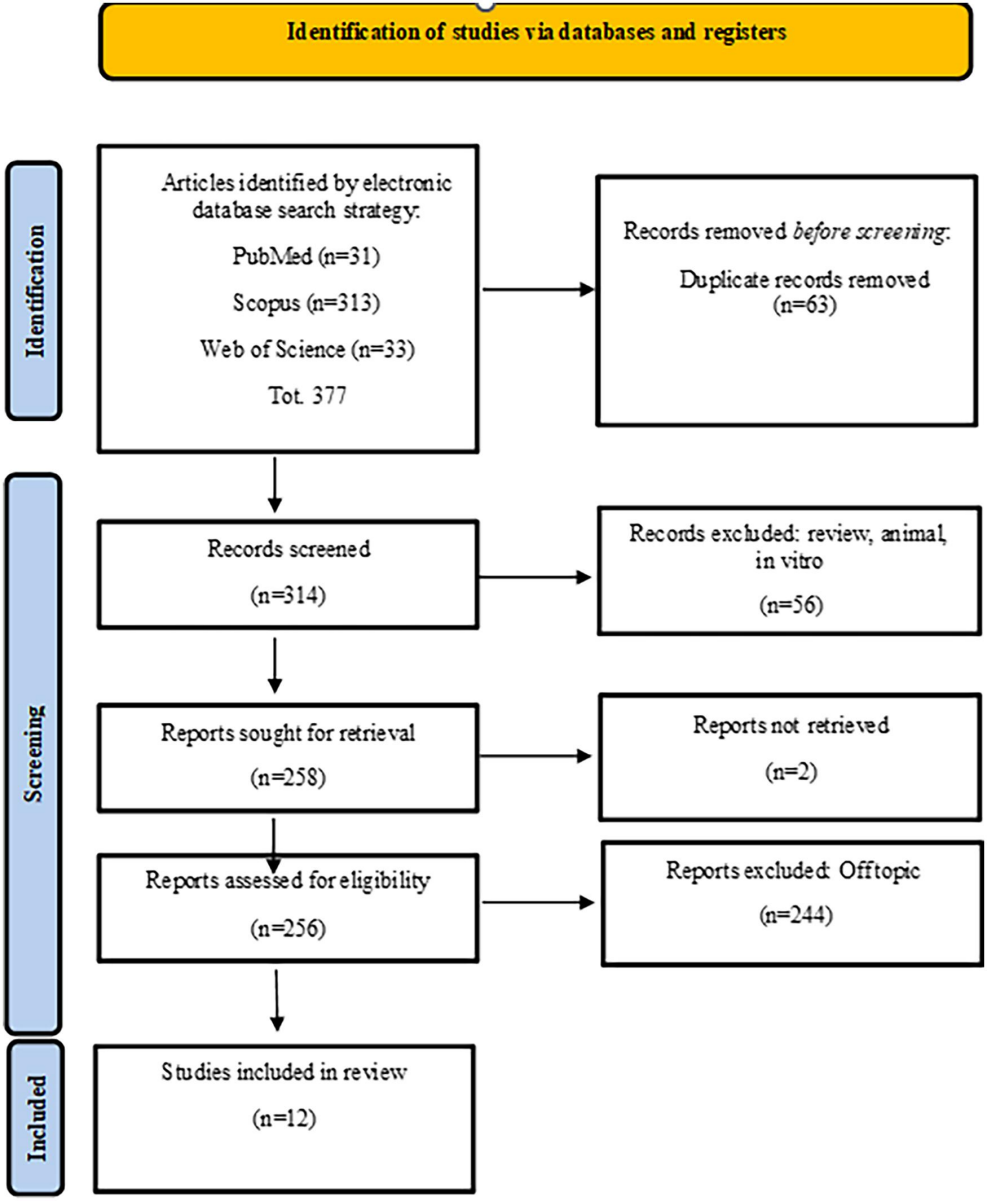
*PRISMA* flow diagram.

The electronic search identified 377 records (PubMed, *n* = 31; Scopus, *n* = 313; Web of Science, *n* = 33). After removal of 63 duplicates, 314 records were screened by title and abstract; 56 records were excluded at the screening stage, 258 records were sought for retrieval, and 2 could not be retrieved. A total of 256 full-text reports were assessed for eligibility; 244 were excluded because they were off-topic, described extra-oral lesions, lacked original case-level information, or provided insufficient clinicopathologic detail. Twelve studies were included in the final qualitative synthesis (Figure 1).

### 3.2 Case description

A 38-year-old man presented with an asymptomatic, slowly enlarging palatal swelling. Intraoral examination showed a well-circumscribed, firm submucosal nodule covered by intact mucosa, without ulceration or spontaneous bleeding. No cervical lymphadenopathy or clinically evident involvement of adjacent structures was detected.

**Figure 2:**
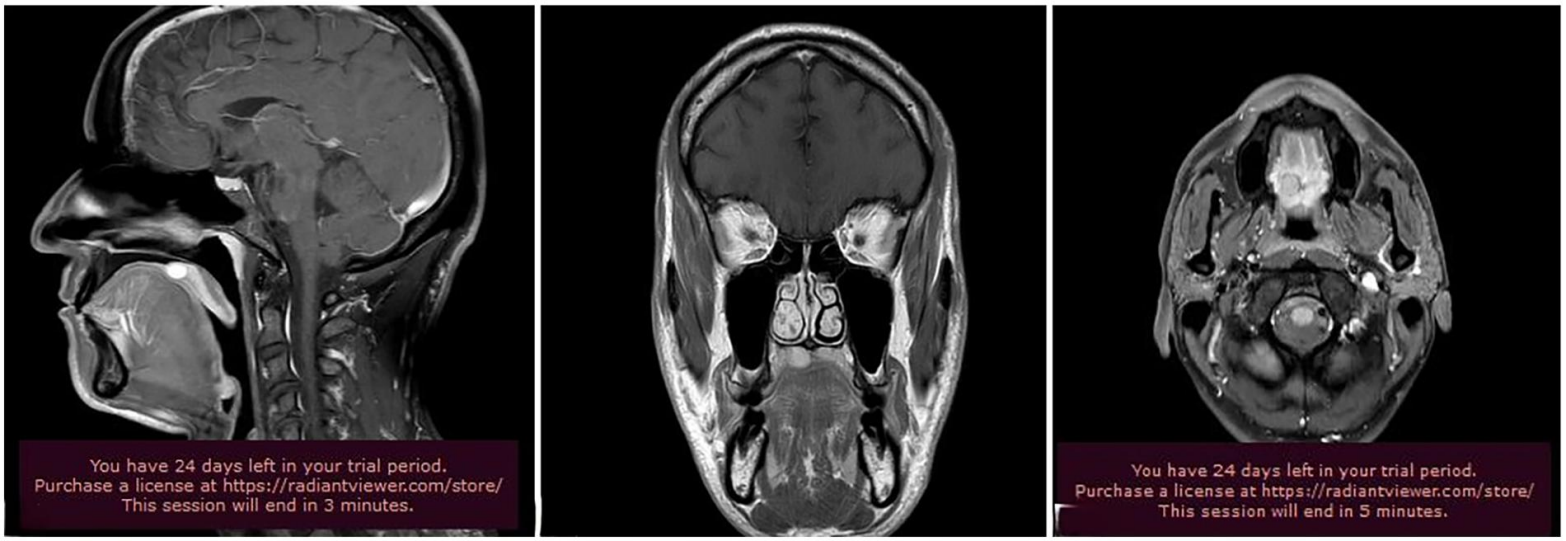
Preoperative magnetic resonance imaging in sagittal **(A)**, coronal **(B)** and axial planes **(C)** showing a well-defined palatal soft-tissue lesion without convincing evidence of cortical bone invasion.

Preoperative magnetic resonance imaging demonstrated a localized, well-defined palatal soft-tissue mass with sharp margins and no convincing evidence of cortical bone invasion. The lesion appeared confined to the palatal soft tissues, without clear radiological signs of aggressive growth or extension into adjacent anatomical compartments. These imaging findings were consistent with a circumscribed lesion and contributed to preoperative assessment of lesion extent and surgical excision planning (Figure 2).

**Figure 3:**
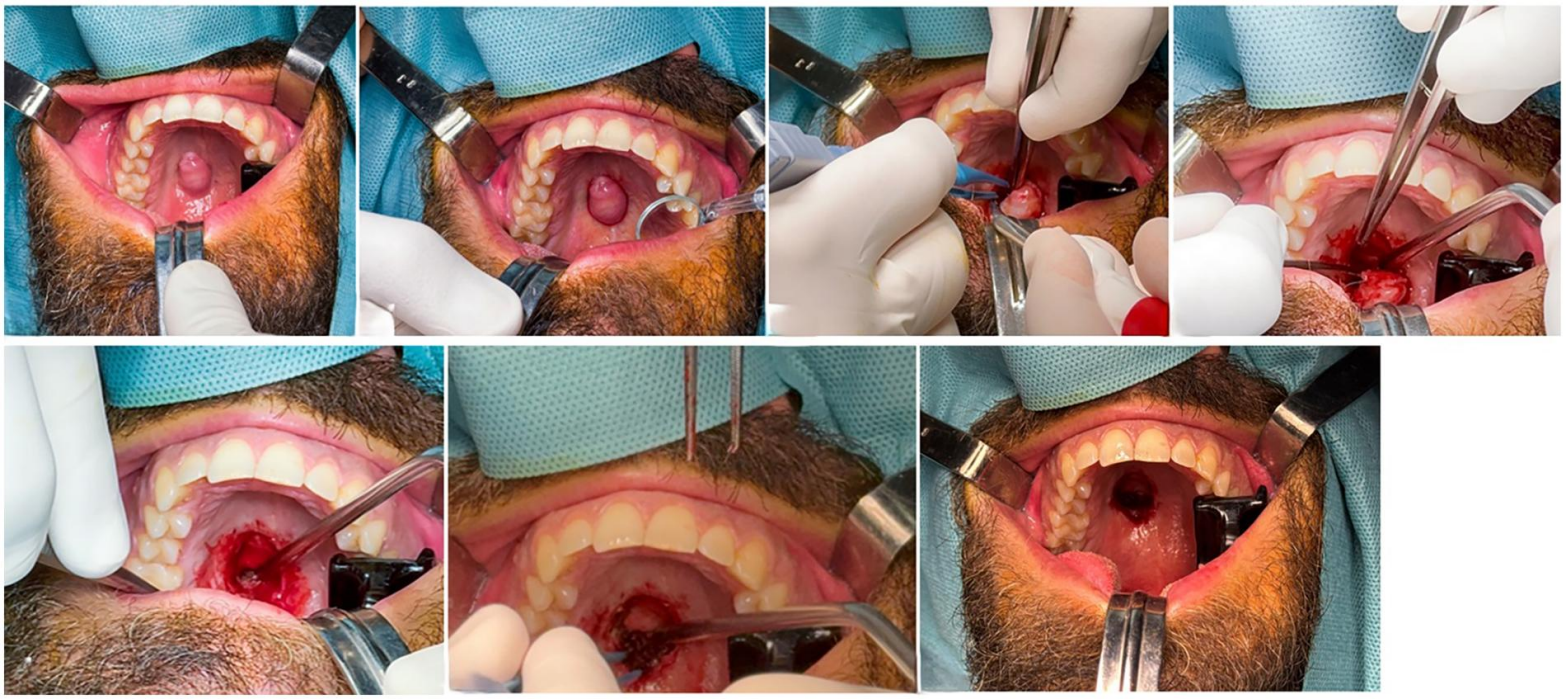
Preoperative and intraoperative sequence of the excision of a palatal lesion consistent with SFT. **(A,B)** Preoperative intraoral views showing a well-defined submucosal nodular lesion involving the hard palate. **(C)** Mucosal and submucosal incision with traction of the lesion. **(D)** Supraperiosteal palatal dissection of the lesion. **(E)** Progressive excision of the lesion, preserving the integrity of the periosteum. **(F)** Hemostasis check and evaluation of the surgical bed after *en bloc* removal. **(G)** Final appearance of the surgical site after coagulation, before application of the hemostatic material.

**Figure 4:**
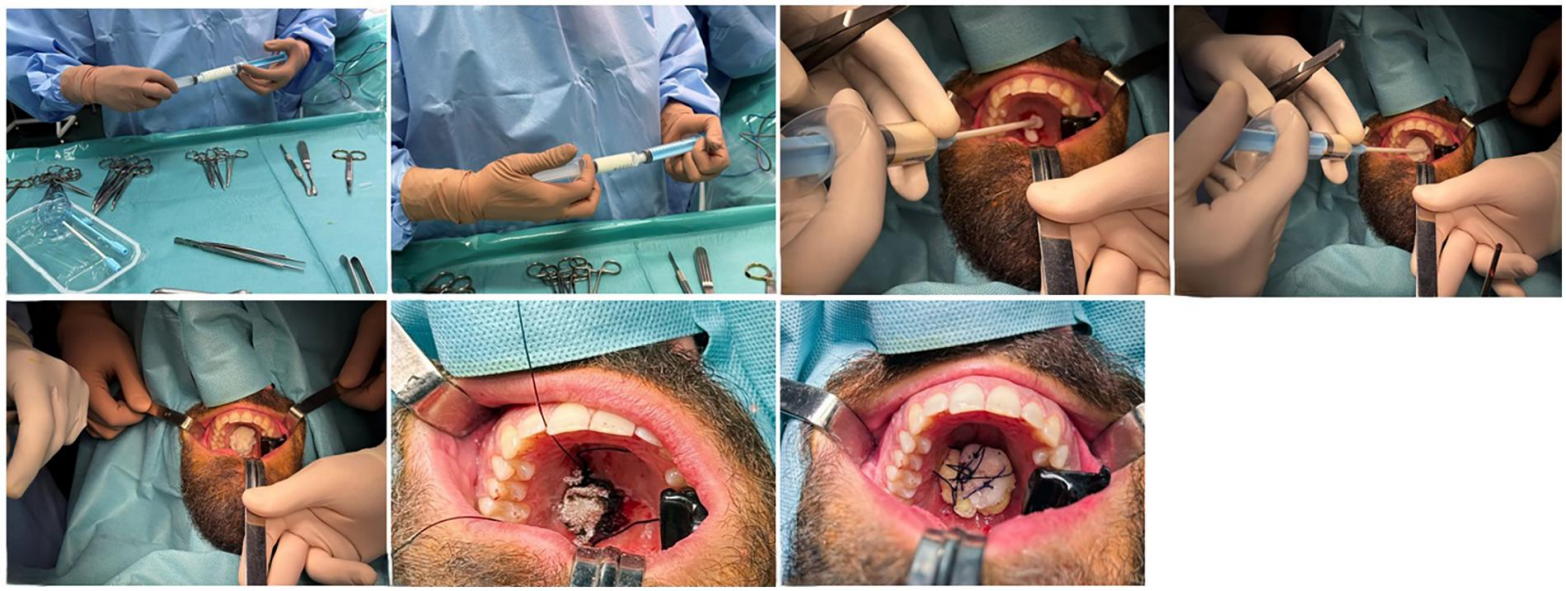
Intraoperative sequence related to hemostatic control and stabilization of the surgical dressing. **(A,B)** Placement of Floseal® on the surgical bed after excision of the lesion, with preservation of the palatal periosteum. **(C,D,E)** The hemostatic material is left in place until complete activation, which occurs in approximately 2 min. **(F)** Placement of four anchoring sutures for subsequent stabilization of the dressing. **(G)** Positioning of a moulage gauze dressing secured with sutures.

An incisional biopsy was performed, followed by complete surgical excision. Surgical management consisted of a circumferential palatal mucosal incision around the lesion, followed by careful blunt and sharp dissection along the cleavage plane under gentle traction. The main steps of the excision procedure are illustrated in Figure 3. The surgical bed was then inspected, irrigated and subjected to meticulous hemostasis; a resorbable hemostatic dressing was positioned and stabilized with sutures to protect the palatal defect and facilitate secondary healing (Figure 4). The lesion was removed *en bloc* as a well-circumscribed nodular mass.

**Figure 5:**
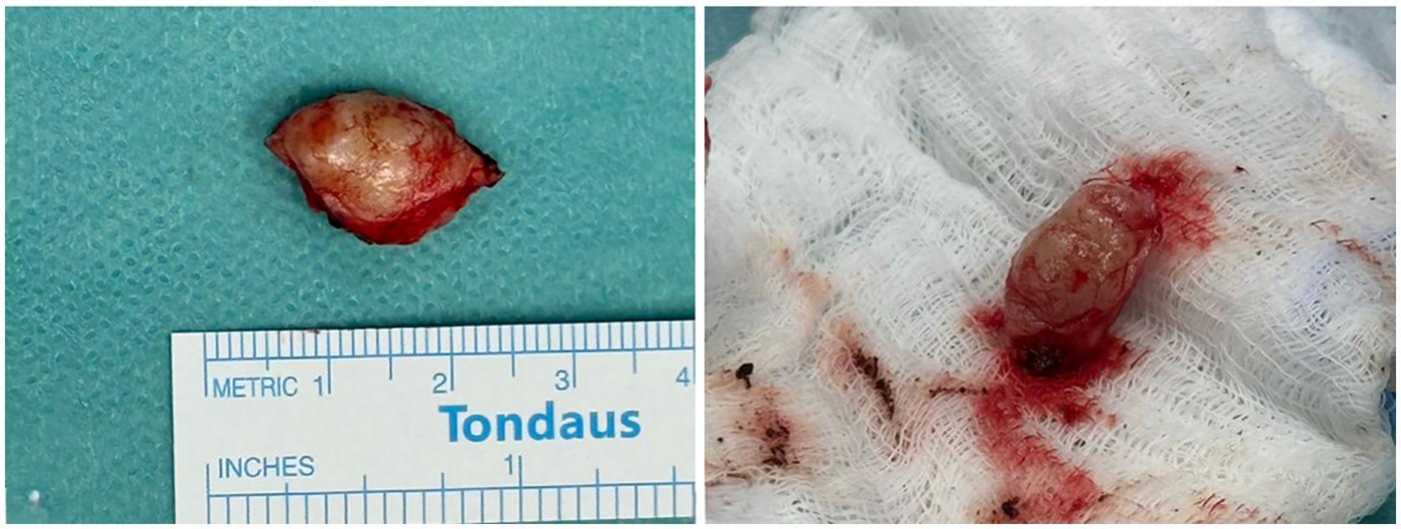
Gross appearance of the excised specimen. **(A)** Fresh surgical specimen measured against a ruler. **(B)** Ovoid, well-circumscribed nodular lesion immediately after removal.

The dimensions of the specimen submitted were 1.7 × 1.2 × 0.8 cm and showed a 0.5 cm whitish area at one end. Histological examination confirmed clear surgical margins (Figure 5).

Formalin-fixed paraffin-embedded tissue sections were stained with hematoxylin and eosin. Immunohistochemical assessment included STAT6, Ki-67 proliferation index (Ki-67) and routine diagnostic markers for spindle-cell lesions, including CD34, CD99, BCL2, Smooth Muscle Actin (SMA), ETS-Related Gene (ERG), S100 and Cyclin-dependent kinase inhibitor 2A (p16).

Microscopically, the lesion consisted of spindle to oval cells arranged in a patternless architecture within a collagenous stroma showing variable hyalinization. Numerous thin-walled branching vessels with a staghorn-like appearance were present. Significant cytologic atypia, necrosis and brisk mitotic activity were not identified.

**Figure 6:**
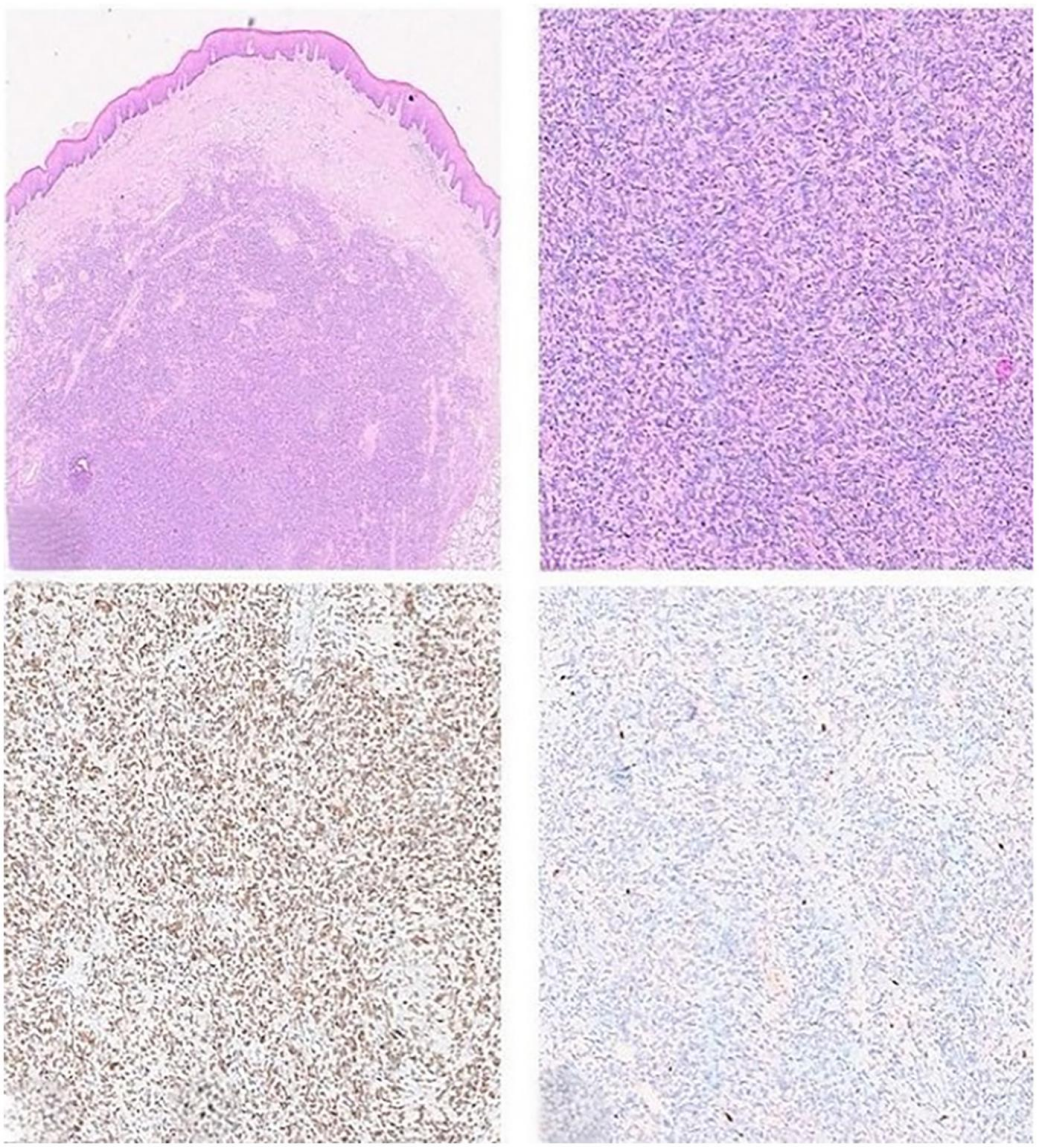
Histopathological and immunohistochemical analysis. **(A)** Low-power view showing a well-circumscribed submucosal spindle-cell proliferation beneath intact oral mucosa. **(B)** Higher magnification showing bland spindle to ovoid cells arranged in a patternless architecture within collagenized stroma with branching vessels. **(C)** Diffuse strong nuclear STAT6 immunoreactivity.

The tumor cells showed diffuse nuclear STAT6 expression and were positive for CD34, CD99 and BCL2, while SMA, ERG, S100 and p16 were negative. The proliferative index was low (Ki-67, approximately 1%–2%). Taken together, the morphologic and immunophenotypic findings supported the diagnosis of SFT (Figure 6).

**Figure 7:**
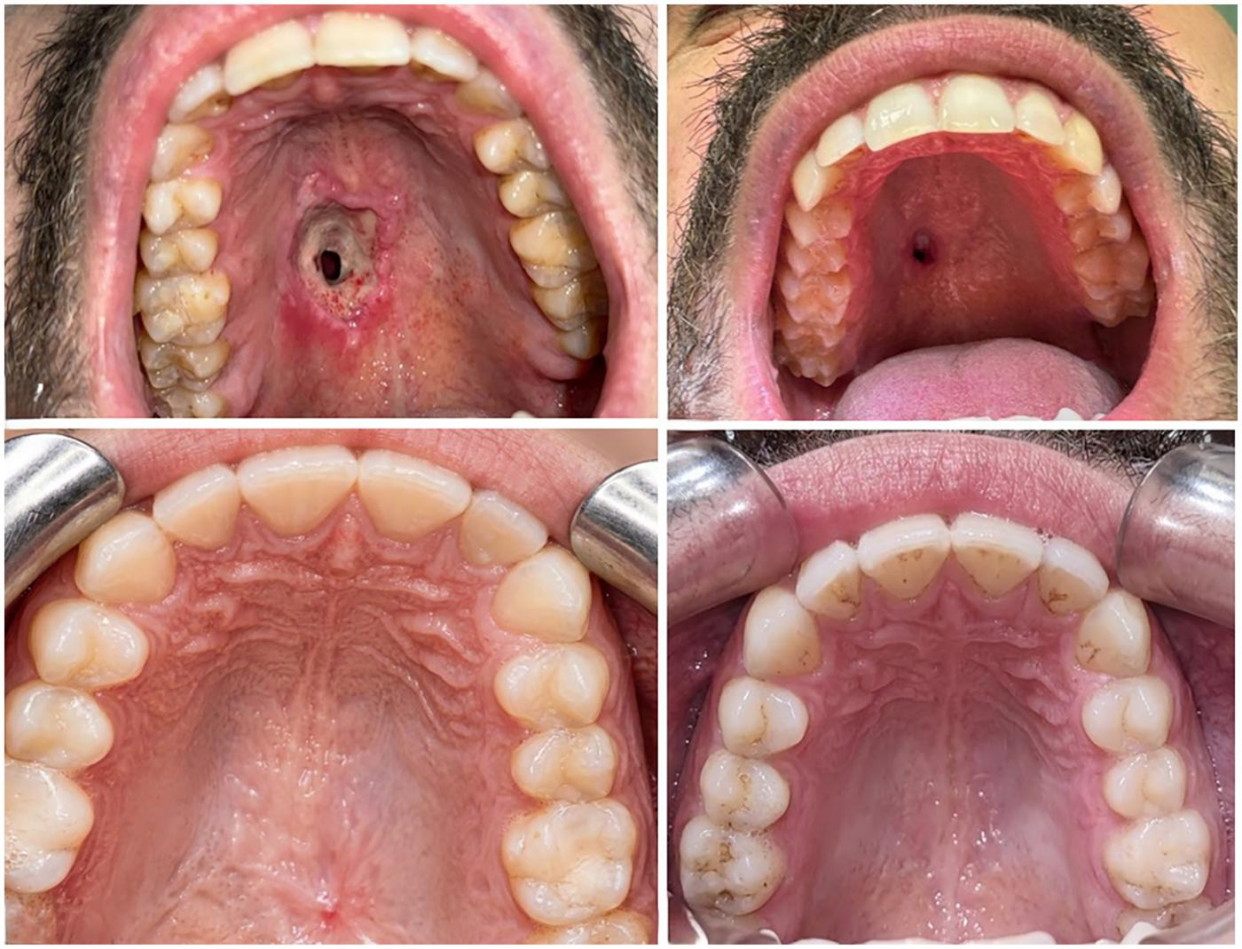
**(A)** early healing of the palatal wound 15 days after excision of the lesion. **(B)** Clinical appearance at 4 weeks after surgery. **(C)** Healing outcome at 2 months. **(D)** Complete healing at 3 months.

Postoperative healing was progressive and uneventful. Early re-epithelialization of the palatal wound was observed at 15 days, and further healing was documented at 4 weeks during serial weekly follow-up visits (dressing changes with Floseal®). At 2 months, the surgical site showed advanced healing and complete restoration of the palatal tissues was achieved at 3 months. An additional clinical follow-up examination was performed 9 months after surgery, showing stable healing of the palatal tissues, with no clinical evidence of local recurrence or functional complaints. Although the currently available follow-up has been extended to 9 months, this interval remains relatively short for SFT. Therefore, the patient will continue long-term surveillance with clinical examinations every 6 months, and radiologic assessment will be performed if symptoms, clinical changes, or suspicion of recurrence arise (Figure 7). Given the short observation period and the possibility of late recurrence in SFT, the patient has been scheduled for long-term surveillance, with clinical examinations every 3 months during the first postoperative year, every 6 months from years 2 to 5, and annually thereafter. Radiologic assessment will be performed in the event of symptoms, clinical changes, or suspicion of local recurrence. The diagnostic work-up and differential diagnosis of oral spindle-cell lesions are discussed in detail below.

### 3.3 Characteristics of the included literature

The 12 included studies consisted of 10 single-case reports and 2 retrospective series, together contributing 30 previously published oral SFTs. The two series accounted for 20 cases: Rodrigues et al. described 13 tumors, predominantly in the buccal mucosa, with additional cases in the tongue and floor of the mouth, whereas Shmuly et al. reported 7 oral SFTs involving the buccal mucosa, labial mucosa and floor of the mouth. The remaining 10 publications each contributed one case involving the buccal mucosa/cheek reported by Raghani et al., Yoon et al. and Trentin-Bordignon et al.; the floor of the mouth reported by Rodrigues et al. and Singh et al.; the tongue reported by Siqueira et al.; the retromolar pad reported by Lotfi et al.; and the intraosseous mandible reported by Mishra et al., Argyris et al. and Fonseca et al.

A wide adult age range was represented, with a slight male predominance overall. Clinically, most lesions were described as painless, slowly enlarging, well-circumscribed nodules or submucosal masses. Larger lesions of the floor of the mouth were associated with tongue displacement, dysarthria or masticatory difficulty, whereas intraosseous mandibular tumors presented radiographically as multilocular or ill-defined radiolucencies with cortical perforation, tooth mobility, root resorption or bone rarefaction.

Histologically, the reviewed lesions were remarkably consistent and typically showed spindle to ovoid cells arranged in a patternless architecture, alternating hypocellular and hypercellular areas, a collagenous to hyalinized stroma, and branching staghorn-like vessels. Cytologic atypia, necrosis and elevated mitotic activity were generally absent in soft-tissue lesions, but increased cellularity, mitotic activity or higher proliferative indices were documented in a minority of cases, particularly in the floor of the mouth and mandible.

Immunohistochemically, STAT6 emerged as the most robust diagnostic marker when evaluated. In the 13-case series by Rodrigues et al. and the 7-case series by Shmuly et al., all tested lesions were STAT6-positive; CD34 was also uniformly positive in both series, while CD99 and BCL2 showed supportive but more variable staining patterns. The case reports broadly confirmed this profile, with most tumors positive for STAT6 and/or CD34 and negative for markers supporting neural, epithelial, myogenic or vascular differentiation.

Complete local excision was the main treatment across the literature. Follow-up was heterogeneous, but most soft-tissue cases remained disease-free after surgery. By contrast, the most clinically relevant adverse outcomes concerned intraosseous mandibular lesions, including recurrence after conservative enucleation and the need for segmental resection in diagnostically challenging bone-based disease (Table 1). When recurrence and adverse outcomes were interpreted in relation to clinicopathologic variables, anatomical site appeared to be the most consistently available and clinically informative factor. Completely excised soft-tissue oral SFTs generally showed a favorable course, whereas intraosseous mandibular lesions represented the subgroup with the greatest concern for diagnostic delay, destructive radiologic presentation, persistence, or recurrence. Surgical margin status, Ki-67 index, and mitotic activity were not uniformly reported across the included studies; therefore, formal comparative analysis of these variables was not feasible. Nevertheless, when available, positive or uncertain margins and increased proliferative activity were considered potential warning features requiring closer follow-up.

**Table 1:** Summary of the studies included in the systematic review***.***

Authors	Type of study	Patients/age/sex/site	Aim of the study	Materials and methods	Conclusions
Argyris P.P. et al. (38)	Case Report + Literature Review	1 case, 54 F Mandible	To report a rare primary intraosseous SFT of the mandible.	Clinical, radiological, and molecular analysis (NAB2-STAT6 fusion).	Intraosseous SFT is extremely rare and can be locally aggressive; STAT6 is essential for a definitive diagnosis.
Fonseca T.C. et al. (39)	Case Report	1 case, 41 F Mandible	To report a recurrence of intraosseous SFT in the mandible.	Clinical, radiological, and immunohistochemistry (IHC) analysis with a 4-year follow-up.	Conservative treatment (enucleation) of intraosseous SFT may lead to late recurrence with infiltrative margins.
Lotfi A. et al. (40)	Case Report	1 case, 49 F Retromolar pad	To describe a rare retromolar-pad SFT and discuss its differential diagnosis.	Histopathologic and IHC analysis (CD34, BCL2).	Understanding various microscopic patterns is vital to prevent unnecessary aggressive treatment.
Mishra S. et al. (41)	Case Report	1 case, 54 F Mandible	To describe an uncommon mandibular Hemangiopericytoma (HPC)/SFT presenting as a jaw lesion.	Clinical examination, imaging, and IHC for CD34 and BCL2.	SFT should be considered in the differential diagnosis of radiolucent lesions of the jaws.
Raghani N. et al. (42)	Case Report	1 case, 60 M Buccal mucosa	To report a buccal mucosal HPC/SFT and emphasize diagnostic difficulty.	Clinical evaluation, excision and histopathologic assessment of a vascular spindle-cell lesion.	Diagnosis is challenging due to vascular patterns; IHC (CD34) is mandatory to rule out other vascular tumors.
Rodrigues M.F.S.D. et al. (43)	Retrospective Clinicopathologic Study	13 cases Various oral sites	To evaluate histomorphology and expression of stem cell markers in oral SFT.	Histopathological analysis and IHC staining for STAT6, CD34, and stem cell markers.	STAT6 is the gold standard for diagnosis; the expression of stem cell markers suggests a specific histogenesis for oral SFTs.
Rodrigues R.M. et al. (44)	Case Report	1 case, 54 F Floor of mouth	To report a floor-of-mouth SFT with long-term follow-up.	Excisional biopsy with histopathologic and IHC (CD34, vimentin, BCL2; negative CD99, S100, SMA, Epithelial Membrane Antigen).	STAT6 is a highly specific marker for oral SFT, facilitating differentiation from other spindle cell tumors.
Singh G. et al. (45)	Case Report	1 case, 48 M Floor of mouth	To report a floor-of-mouth SFT with high proliferative index.	Clinical and radiologic evaluation with histopathology, immunohistochemistry and Ki-67 assessment	A high Ki-67 index may suggest more aggressive behavior, necessitating closer clinical monitoring**.**
Siqueira J.M. et al. (33)	Case Report	1 case, 28 M Tongue	To report a rare SFT in the tongue.	Clinical data review, histological diagnosis, and 24-month follow-up.	SFT of the tongue is rare; complete surgical removal results in a good prognosis.
Shmuly T. et al. (46)	Retrospective Clinical Study	7 cases Various oral sites	To analyze clinico-pathological features and long-term follow-up of oral SFT.	Chart review, histopathology and immunohistochemistry including CD34, CD99, BCL2 and STAT6.	Oral SFT is usually benign with an excellent prognosis if completely excised; long-term follow-up is essential.
Trentin-Bordignon N.C. et al. (47)	Case Report	1 case, 25 M Buccal mucosa	To describe the diagnostic challenges of oral SFT.	Excisional biopsy and immunohistochemistry for STAT6, CD34, beta-catenin and BCL2.	SFT can mimic malignant neoplasms; IHC markers like STAT6 provide a definitive diagnosis.
Yoon C.M. et al. (48)	Case Report	1 case, 50 M Buccal mucosa	To report a rare SFT in the buccal cheek mucosa with follow-up.	Total excision under local anesthesia and postoperative monitoring for 5 years.	No recurrence was noted after 5 years; total surgical excision is the curative treatment.

### 3.4 Methodological quality assessment

The included evidence was inherently limited by the predominance of single case reports and small retrospective case series. Most studies adequately reported patient demographics, lesion site, histopathological findings, and immunohistochemical profile. However, recurrent limitations included incomplete description of imaging findings, variable reporting of surgical margins, heterogeneous immunohistochemical work-up, and inconsistent follow-up duration. The two retrospective series provided broader clinicopathologic information, but remained limited by retrospective design, relatively small sample size, and possible selection bias. Overall, the methodological quality of the included literature was judged as moderate, with higher concern in reports providing limited diagnostic characterization or insufficient follow-up. These limitations support cautious interpretation of the available evidence and justify the descriptive nature of the present synthesis (Table 2).

**Table 2:** Methodological appraisal of the included studies according to JBI critical appraisal tools.

Authors	Study design	Appraisal tool	Main concerns	Methodological concern
Argyris P.P. et al. (38)	Case report	JBI Case Report Checklist	Single-case design; limited generalizability	Moderate concern
Fonseca T.C. et al. (39)	Case report	JBI Case Report Checklist	Single-case design; recurrence reported, but limited external validity	Moderate concern
Lotfi A. et al. (40)	Case report	JBI Case Report Checklist	Limited immunohistochemical characterization; follow-up details not fully comprehensive	Moderate concern
Mishra S. et al. (41)	Case report	JBI Case Report Checklist	Limited diagnostic panel; possible diagnostic overlap with related entities	High concern
Raghani N. et al. (42)	Case report	JBI Case Report Checklist	Sparse diagnostic work-up and outcome reporting	High concern
Rodrigues M.F.S.D. et al. (43)	Retrospective clinicopathologic study	JBI Case Series Checklist	Retrospective design; possible selection bias; heterogeneous case documentation	Moderate concern
Rodrigues R.M. et al. (44)	Case report	JBI Case Report Checklist	Single-case design, despite adequate clinicopathologic description	Moderate concern
Singh G. et al. (45)	Case report	JBI Case Report Checklist	Limited external validity; variable follow-up detail	Moderate concern
Siqueira J.M. et al. (33)	Case report	JBI Case Report Checklist	Single-case design; limited generalizability	Moderate concern
Shmuly T. et al. (46)	Retrospective clinical study	JBI Case Series Checklist	Retrospective design; possible selection bias; heterogeneous follow-up	Moderate concern
Trentin-Bordignon N.C. et al. (47)	Case report	JBI Case Report Checklist	Single-case design; limited external validity	Moderate concern
Yoon C.M. et al. (48)	Case report	JBI Case Report Checklist	Single-case design despite adequate follow-up	Moderate concern

## 4 Discussion

Oral SFT remains an uncommon clinicopathologic entity, and current evidence is derived mainly from case reports and small institutional series (49–53). Even so, the 12 studies included in this review provide a coherent message: most oral SFTs are circumscribed, indolent soft-tissue lesions, but a small subset, especially intraosseous mandibular tumors, can mimic aggressive disease radiologically and may recur after limited surgery (52, 54–57).

### 4.1 Clinical presentation and anatomical distribution

The predominance of buccal mucosal lesions in the present review is not incidental (58–61). Rodrigues et al. and Shmuly et al. showed that the buccal mucosa was the most frequently involved site in their retrospective cohorts, and this pattern is reinforced by the single-case reports of Raghani et al., Yoon et al. and Trentin-Bordignon et al. (42, 43, 46–48). Floor-of-mouth tumors reported by Rodrigues et al. and Singh et al. tended to be larger and more symptomatic, often causing tongue displacement, dysarthria or masticatory discomfort (44, 45). Siqueira et al. documented tongue involvement, Lotfi et al. highlighted the retromolar pad as an unusual site, and the current palatal case further expands the soft-tissue spectrum of this tumor within the oral cavity (33, 40, 62–66).

### 4.2 Histopathological features

From a morphologic perspective, the literature consistently confirms the diagnostic importance of the triad seen in the present case: patternless spindle-cell proliferation, collagenized or hyalinized stroma, and branching staghorn-like vasculature (67–71). However, the included studies also show why misclassification remains possible (72, 73). Variable cellularity, myxoid change, giant cells, skeletal muscle entrapment, epithelioid areas and increased mitotic counts were each documented in at least some reports (74–77). These features broaden the histologic spectrum of oral SFT and explain its overlap with myofibroma, schwannoma, neurofibroma, leiomyoma, salivary gland neoplasms and other hemangiopericytoma-like spindle-cell lesions (78–81). Immunohistochemistry remains essential for resolving the differential diagnosis of oral spindle-cell lesions (82–85). Across the reviewed studies, nuclear STAT6 expression represented the most useful confirmatory marker for SFT, whereas CD34, CD99, and BCL2 were supportive but less specific (86–89). Therefore, once morphology raises suspicion for SFT, STAT6 should be prioritized within a broader immunohistochemical panel designed to exclude neural, myogenic, epithelial, melanocytic, and vascular tumors (90–98). 4.3 Biological Behavior and Risk Stratification.

The biological behavior of oral SFT is usually indolent, but the current review does not support complacency (99–103). Soft-tissue lesions excised completely generally had excellent outcomes, with no recurrence reported in the Shmuly series and in several individual reports, including those of Yoon et al., Rodrigues et al., Siqueira et al. and Trentin-Bordignon et al. (33, 44, 47, 48, 104, 105). At the same time, histologic blandness does not automatically exclude risk (106–110). Rodrigues et al. found more than 4 mitoses per 10 high-power fields in 2 of 13 tumors, and Singh et al. described a floor-of-mouth lesion with a Ki-67 index of 25% and 5–7 mitoses per 10 high-power fields despite the absence of frank necrosis or infiltrative margins (43, 45, 111–114).

The strongest warning signal in the recent literature comes from mandibular intraosseous disease (115–118). Mishra et al. reported a jaw lesion that clinically resembled odontogenic pathology, whereas Argyris et al. documented a fusion-confirmed primary intraosseous SFT with cortical perforation and long-standing growth that required segmental mandibulectomy (38, 41, 119, 120). Fonseca et al. then demonstrated that recurrence may develop four years after conservative enucleation when margins are compromised (121–124). These mandibular reports make it clear that site matters: bone-based SFTs appear to carry a higher potential for diagnostic delay, more destructive radiologic presentation and local persistence or recurrence (125–129). Accordingly, treatment planning should not be guided by nomenclature alone but by the combination of site, margin status, growth pattern and proliferative features (130, 131). For most well-circumscribed soft-tissue oral SFTs, conservative complete excision is appropriate (132, 133). However, lesions with intraosseous extension, positive margins or increased mitotic/proliferative activity deserve closer multidisciplinary evaluation and a lower threshold for more definitive surgery or prolonged surveillance (134, 135). Based on these observations, a practical risk-stratified management framework may be proposed for oral SFT. This framework is not intended as a validated prognostic model, but as a pragmatic clinical synthesis derived from the available case-based evidence (Table 3).

**Table 3:** Practical risk-stratified management framework for oral SFT.

Risk group	Clinicopathologic features	Suggested management
Low risk	Soft-tissue lesion; well-circumscribed growth; complete excision; negative margins; low Ki-67; absence of necrosis, atypia, or increased mitotic activity	Complete local excision with margin assessment; clinical follow-up
Intermediate risk	Close or uncertain margins; larger lesion; floor-of-mouth or functionally complex site; mildly increased mitotic/proliferative activity	Multidisciplinary review; consider re-excision if feasible; closer follow-up
High risk	Intraosseous presentation; positive margins; recurrence; destructive radiologic pattern; elevated Ki-67 or mitotic activity; atypia or necrosis	Multidisciplinary management; complete resection when feasible; long-term clinical and radiologic follow-up

### 4.3 The importance of long-term follow-up

Follow-up remains one of the weakest and most heterogeneous aspects of the available literature (136–138). Observation periods ranged from very short intervals to 74 months in the Shmuly series, 5 years in the buccal mucosa case of Yoon et al., and 8 years in the floor-of-mouth case of Rodrigues et al. (44, 46, 48, 85, 139, 140). Because late recurrence was documented by Fonseca et al. after a four-year gap in care, the absence of early postoperative disease cannot be considered definitive (39, 141–143). A minimum follow-up of 5 years appears reasonable for oral SFT, while bone-based, margin-positive or proliferatively active lesions should be monitored even longer, ideally with periodic clinical and radiologic assessment (144, 145). A limitation of the present report is the still relatively short follow-up currently available, although it has been extended to 9 months after surgery. Therefore, the absence of recurrence at this stage should not be interpreted as definitive evidence of long-term disease control. Continued surveillance is planned every 6 months, with additional radiologic assessment in the event of symptoms, clinical changes, or suspicion of recurrence (146, 147).

### 4.4 Differential diagnosis and diagnostic workflow

At initial clinical presentation, oral SFT may mimic several benign or malignant lesions because it usually appears as a slow-growing, well-circumscribed submucosal mass covered by intact mucosa. In palatal or oral soft-tissue locations, the main clinical differential diagnoses include minor salivary gland tumors, such as pleomorphic adenoma, canalicular adenoma, mucoepidermoid carcinoma, and adenoid cystic carcinoma; reactive or fibrous lesions; vascular lesions; benign peripheral nerve sheath tumors; smooth-muscle tumors; and other mesenchymal proliferations. In intraosseous mandibular presentations, odontogenic cysts and tumors should also be considered. Histologically, the differential diagnosis includes schwannoma, neurofibroma, myofibroma, leiomyoma or leiomyosarcoma, spindle-cell carcinoma, melanoma, vascular tumors, and other soft-tissue sarcomas. Because morphology alone may be insufficient, diagnosis should rely on integration of clinical findings, imaging, histopathology, and a targeted immunohistochemical panel. Diffuse nuclear STAT6 expression in the appropriate morphologic context strongly supports SFT, while molecular confirmation of NAB2–STAT6 fusion may be useful in equivocal cases. A proposed diagnostic workflow for oral spindle-cell lesions suspicious for SFT is summarized in (Table 4).

**Table 4:** Proposed diagnostic workflow for oral spindle-cell lesions suspicious for solitary fibrous tumor.

Step	Diagnostic phase	Key elements
1	Initial clinical presentation	Persistent oral submucosal mass or suspected spindle-cell lesion
2	Clinical assessment	Evaluation of site, size, growth rate, mucosal changes, bleeding, pain, neurologic signs, and cervical lymph nodes
3	Site-directed imaging	MRI for soft-tissue lesions; CT/CBCT for suspected bone involvement; Doppler or angiographic assessment if a vascular lesion is suspected
4	Biopsy	Incisional biopsy for large or deep lesions; excisional biopsy for small, well-circumscribed lesions
5	Histopathological evaluation	Patternless spindle-cell proliferation; collagenized or hyalinized stroma; branching staghorn-like vessels
6	Initial immunohistochemical panel	STAT6, CD34, CD99, BCL2; exclusion markers including S100/SOX10, SMA/desmin, cytokeratins/p63, ERG/CD31
7	Diagnostic interpretation	Compatible morphology plus diffuse nuclear STAT6 expression supports the diagnosis of SFT; NAB2–STAT6 molecular testing may be considered in equivocal cases
8	Treatment and follow-up	Complete surgical excision with margin assessment; risk-adapted long-term surveillance

## 5 Conclusion

This systematic review of 12 recent studies, together with the present palatal case, shows that oral SFT is a rare but morphologically recognizable fibroblastic neoplasm whose diagnosis relies on careful clinicopathologic correlation and confirmatory immunohistochemistry. The reviewed literature demonstrates that most lesions arise in soft tissue, especially the buccal mucosa, and follow a favorable course after complete excision. Nevertheless, oral SFT is not uniformly innocuous: lesions with intraosseous mandibular presentation, positive margins, or increased proliferative activity can behave more aggressively or recur. The present case supports these conclusions by illustrating the classic microscopic phenotypeof oral SFT, a confirmatory immunophenotype, and a low Ki-67 index.Oral SFT should therefore be included in the differential diagnosis of oral spindle-cell tumors. Complete excision with margin assessment should be pursued whenever feasible, and long-term follow-up should be adapted to risk-related features such as intraosseous presentation, positive or uncertain margins, recurrence, or increased proliferative activity. However, the present case remains limited by relatively short-term follow-up, despite the absence of recurrence at 9 months, and ongoing long-term surveillance is required.

## Data Availability

The raw data supporting the conclusions of this article will be made available by the authors, without undue reservation.
